# The Psychedelic Debriefing in Alcohol Dependence Treatment: Illustrating Key Change Phenomena through Qualitative Content Analysis of Clinical Sessions

**DOI:** 10.3389/fphar.2018.00132

**Published:** 2018-02-21

**Authors:** Elizabeth M. Nielson, Darrick G. May, Alyssa A. Forcehimes, Michael P. Bogenschutz

**Affiliations:** ^1^Department of Psychiatry, NYU School of Medicine, New York University, New York, NY, United States; ^2^Department of Psychiatry & Behavioral Sciences, Johns Hopkins University School of Medicine, Baltimore, MD, United States; ^3^Train for Change, Inc., Phoenix, AZ, United States

**Keywords:** alcoholism, psilocybin, motivational interviewing, hallucinogens, psychedelic assisted therapy, ego-dissolution, addiction treatment, psychotherapy

## Abstract

Research on the clinical applications of psychedelic-assisted psychotherapy has demonstrated promising early results for treatment of alcohol dependence. Detailed description of the content and methods of psychedelic-assisted psychotherapy, as it is conducted in clinical settings, is scarce.

**Methods:** An open-label pilot (proof-of-concept) study of psilocybin-assisted treatment of alcohol dependence (NCT01534494) was conducted to generate data for a phase 2 RCT (NCT02061293) of a similar treatment in a larger population. The present paper presents a qualitative content analysis of the 17 debriefing sessions conducted in the pilot study, which occurred the day after corresponding psilocybin medication sessions.

**Results:** Participants articulated a series of key phenomena related to change in drinking outcomes and acute subjective effects of psilocybin.

**Discussion:** The data illuminate change processes in patients' own words during clinical sessions, shedding light on potential therapeutic mechanisms of change and how participants express effects of psilocybin. This study is unique in analyzing actual clinical sessions, as opposed to interviews of patients conducted separately from treatment.

## Introduction

Psychedelic-assisted psychotherapy is a growing field and includes treatments such as psilocybin-assisted treatment of addictions and depression, both of which are currently in phase 2 clinical trials. The efficacy of one such psychedelic-assisted therapy, psilocybin-assisted treatment of alcohol dependence, is currently being tested in an FDA-approved phase 2 clinical trial (NCT02061293), having demonstrated safety and potential for effectiveness in an open-label pilot study (NCT01534494) with a similar design. The treatment in both studies consists of 12 weekly psychotherapy sessions with two psilocybin medication sessions at weeks 4 and 8. Participants meet with two therapists who each have a specific role. In the pilot, one therapist provided Motivational Enhancement Therapy (MET) therapy, a psychosocial treatment based on motivational interviewing (MI), designed to build motivation through evoking the patient's reasons for change and strengthening skills to support the patient's goals around changes in alcohol use. The other therapist focused on helping the patient prepare for and integrate the psychedelic experience (Bogenschutz and Forcehimes, 2016). Both therapists were also present for the preparation sessions and psychedelic medication administration sessions as well as the debriefing sessions, which took place the following day after the acute effects have worn off. Therapist roles and the structure of therapy remain largely the same in the phase 2 trial, with the addition of some cognitive behavioral interventions introduced after the first medication session. Debriefing sessions, nearly identical in both studies, are an integral part of the series of non-drug psychotherapy sessions patients receive.

Bogenschutz and Forcehimes (2016) describe the debriefing as a session in which “the patient provides a full account of the experience, relatively soon (hours to a few days) after the experience, in as much detail as possible, and is asked to reflect on the significance of the experience, and to describe any persisting positive or negative effects that are apparent” (p. 10). In most clinical research the study protocol and/or therapy manual describe debriefing sessions and outline some required content, such as assessment of mental status and screening for adverse reactions. The bulk of the debriefing session is devoted to an open-ended discussion of the patients' experience (Bogenschutz and Forcehimes, 2016). This format offers a unique opportunity to explore the patient's experience.

Although debriefing session content, structure, and timing may vary between research studies, the sessions that receive this title are typically the first ones held after a psychedelic session and focus on giving the participant an opportunity to describe their experience. Psilocybin experiences are by their very nature quite different from ordinary states of consciousness. They have been described in the literature as both beyond words and transcending time and space (MacLean et al., 2012). Since the encoding of experience into long-term memory involves language and the assimilation of new experiences with existing knowledge, these experiences may be more difficult to remember and recall later than others that are more similar to what the individual already knows. Discussion of psychedelic experiences during a debriefing session may help participants benefit from them by consolidating memories of the experience, processing emotions, and articulating insights.

Where the use of classic hallucinogens for therapy is concerned, early research was largely conducted with LSD, a serotonergic psychedelic similar to psilocybin. Most methods employed with one compound have and/or can be used with the other. Leuner (1967) discussed two early therapeutic models for psychedelic-assisted treatment: psychedelic and psycholytic therapy. Psychedelic therapy involves using high doses of a psychedelic to attain “cosmic-mystic experiences, oneness and ecstatic joy,” (p. 102) without the context of extensive, ongoing psychotherapy. Goals are usually change in target behaviors, i.e., drinking, through one single, overwhelming experience. Psycholytic therapy, on the other hand, involves low-medium doses to produce a dreamlike, regressed state, during which psychoanalytic psychotherapy takes place, as part of a longer course of psychotherapy. Leuner considered psycholytic therapy appropriate for indications such as psychosomatic cases, paraphilic disorders, border-line (psychosis), but ruled out alcoholism as treatable with this method (Leuner, 1967). Building on Leuner's comparison, Caldwell (1968) cautioned that the “the value of the transcendental, mystical, or peak experience is open to considerable debate” (p. 119). While conceding that early follow up-data from psychedelic therapy with LSD for alcoholics were promising, Caldwell found it difficult to imagine a lifetime of conditioning would be changed in just a few hours. The present study's attention to psychotherapy surrounding the psilocybin sessions demonstrates a recognition of the idea that change as a result of the overwhelming, peak experiences of traditional psychedelic therapy can be consolidated or enhanced through psychotherapy.

In alcoholism treatment specifically, some researchers have had positive outcomes using ketamine—a dissociative anesthetic that produces subjective states similar to those produced by psychedelics—in combination with other drugs such that patients experience negative emotional states in combination with the psychedelic-like effects (Krupitsky et al., 1992). This method, called Affective Contra-Attribution (ACA), has some overlap with the methods used in the present study in that in includes preparatory and integration psychotherapy flanking the ketamine session. The ACA method is different, however, in that patients are presented with alcohol while experiencing negative affective states during the ketamine sessions in order to build negative associations with alcohol. Also, unlike the present model, ACA integration sessions take place in a group format. Krupitsky and Grinenko (1997) later moved away from the ACA model to study ketamine psychedelic therapy (KPT), which does not include the induction of negative emotional states or presenting the patient with alcohol during the session, and includes engaging the patient in psychotherapy during the ketamine session. Outcomes of KPT studies suggest that it can increase the effectiveness of conventional treatment for alcohol dependence (Krupitsky and Grinenko, 1997) and produce greater rates of abstinence and reduction of craving for heroin in people with opioid use disorder (Krupitsky et al., 2002).

In the early 1950's, LSD researchers Hoffer and Osmond proposed that psychedelic therapy with LSD could induce an experience akin to *delirium tremens*, a dangerous and extreme consequence of alcohol withdrawal, which many recovered alcoholics cited as having been the point at which they realized the need to change their drinking (Hoffer, 1967). Given that the delirium tremens could be deadly, the ability to induce a subjectively similar yet physically safer state that could potentially motivate change was intriguing. Over the next 10 years, this theory gave rise to 11 studies with a total of 311 patients, 145 (46%) of which were considered “much improved” at follow up. Although research standards of that time were substantially less rigorous than they are today, this improvement was striking when compared to the 13.7% improvement rate in the comparison sample (*N* = 80) that received standard treatment (Hoffer, 1967). Hoffer described the settings for LSD administration in the 11 studies as “comfortable, relaxed and the subjects were allowed to think, feel and meditate on the insights they acquired” (1967, p. 353). Although the present study did not attempt to induce a *delirium tremens-like* experience, the emphasis on creating a comfortable, relaxing setting can be traced to this early work.

Levine and Ludwig (1967) developed the *hypnodelic* technique to maximize the chances of therapeutic success in treating alcoholics with LSD by “directing the patients' attention to his present problems and trying to get him to understand them in terms of his previous conflicts” (pp. 549–550). Seeing little potential benefit in having their patients experience hallucinations and states of bliss, and seeking to capitalize on the potential of LSD to enhance their patients' self-knowledge and insight, they combined LSD administration with hypnosis. In this method, patients were hypnotized after ingesting LSD, but before it took effect, such that patients experienced the LSD and hypnosis simultaneously. During the LSD/hypnosis sessions, therapists actively engaged the patient in psychotherapy to explore problems, encourage the expression and release of previously repressed emotions (abreaction), and encourage new insight through interpretations (Levine and Ludwig, 1967). While the use of hypnosis distinguishes this model, the engagement in active psychotherapy during a psychedelic session is akin to the psycholytic model described above, and goals of exploring problems, abreaction, and gaining insight are echoed in the present study.

More recently, in treatment of cancer-related anxiety with psilocybin, psychodynamic therapies such as logotherapy and meaning-making therapy have been used as the psychotherapeutic models (Ross et al., 2016). Other modern addiction treatment protocols such as psilocybin-assisted smoking cessation have used a cognitive behavioral therapy (CBT) model (Johnson et al., 2014). A study of psilocybin-assisted treatment of depression currently under development will use Acceptance and Commitment Therapy, a third-wave CBT approach that combines traditional CBT and mindfulness practices (Guss, 2017, Personal Communication). While the choice of the MET model for the present study was based on factors such as feasibility and demonstrated efficacy in treating addiction, it is not the only approach that could be combined with psychedelic preparation and integration sessions to create a psychedelic-assisted treatment protocol.

Undergoing psilocybin-assisted treatment for alcohol dependence in a clinical setting is a rare experience. Individuals participating in this treatment do not have the support of a group of peers who have also been through the same treatment, in which they can comfortably discuss the experiences and changes they attribute to it. Friends, family, and peers may respond with a variety of attitudes including support, curiosity, disbelief, or even negative judgments. This set of conditions, in addition to general stigma surrounding addiction diagnosis and treatment (Luoma et al., 2007), make it especially important that the individual have ample time to discuss and process the experience with their therapists, who form a temporary yet invaluable support network. With its open-ended format and focus on the participant's experience in the medication session, the debriefing session is an example of how this support is provided. As psilocybin-assisted therapy for alcohol dependence is still in early development as a clinical practice, it is unknown how patients describe key phenomena related to behavioral change and symptom improvements. The purpose of this investigation is to explore the ways in which patients talk about change-related phenomena during post-medication debriefing sessions.

## Method

Ten participants meeting DSM-IV-TR criteria for alcohol dependence were enrolled in an open-label pilot study of psilocybin-assisted treatment of alcohol dependence (NCT01534494), the primary outcome of which was previously published (Bogenschutz et al., 2015). All participants provided written informed consent approved by the IRB of the University of New Mexico, which included consent for transcripts of their sessions to be analyzed for the present project. Participants were excluded if they had exclusionary medical or psychiatric conditions; family history of schizophrenia, bipolar disorder, or suicide; cocaine, psychostimulant, or opioid dependence; or history of using hallucinogens more than 10 times (or at all in the past 30 days). As noted above, the treatment protocol included 14 treatment sessions over 12 weeks, including two psilocybin medication sessions at weeks four and eight, and 12 non-drug psychotherapy sessions, two of which were debriefing sessions.

Psilocybin was given orally in a dose of 0.3 mg/kg for the first psilocybin session and 0.4 mg/kg for the second session (second session could be held at 0.3 mg/kg if there were strong effects in the first session, if the participant did not want to increase, or if the clinical team felt an increase would be clinically contraindicated). No previously published studies had established a dose range of psilocybin for alcohol dependence, however, an analysis of published psilocybin research with healthy volunteers and doses of LSD used historically in the treatment of alcoholism led to the conclusion that a dose range of 0.29–0.43 mg/kg of psilocybin “seems to be an appropriate starting point for addiction treatment studies” (Bogenschutz, 2013, p. 20). Full details of the study design, participant demographics, and treatment model are provided elsewhere (Bogenschutz et al., 2015; Bogenschutz and Forcehimes, 2016).

Each participant had a debriefing session with both therapists on the day after each psilocybin medication session. Debriefing sessions included examination of the participant's mental status—including thought process, thought content, perception, and orientation—and open-ended discussion of the patients' experience. We conduced qualitative content analysis (Schreier, 2014) of transcripts from all 17 debriefing sessions conducted as part of the pilot study in order to illustrate how patients talk about change-related phenomena in this unique context. The 17 sessions included in this analysis represent the complete set of debriefing sessions conducted during the study as 10 patients participated, with seven completing two sets of psilocybin and debriefing sessions and three completing only one set of psilocybin and debriefing sessions. Because our analysis is of debriefing sessions that took place as part of a series of psychotherapy sessions as opposed to structured qualitative interviews conducted apart from the therapeutic intervention by an independent (non-clinician) interviewer, we draw our examples from statements offered spontaneously by patients in the context of therapy. All names used in this report are pseudonyms and text has been edited to remove vocalized pauses and filler utterances.

### Measures

The pilot study included multiple, repeated measures of patients' mood, mental status, physical health, vital signs, and drinking outcomes, all of which are detailed in Bogenschutz et al. (2015). The 43 item Mystical Experience Questionnaire (MEQ) was used to determine the presence and strength of experiences that are similar to mystical experiences which may occur spontaneously or in a religious context, including those in which a psychedelic compound is part. In order to delineate such experiences when they occur in secular, research settings, we describe them here as mysticomimetic—mimicking mystical experiences, and perhaps phenomenologically indistinguishable, but different based on the context in which they occur. Based on Stace's (1961) operational definition of mystical experiences (Barrett et al., 2015), the MEQ was originally developed in the 1960's by Pahnke (1963, 1969) to measure mysticomimetic experiences during psychedelic drug administration sessions in research settings, with later refinement and validation by Richards et al. (1977). A recent factor analysis and further validation (MacLean et al., 2012; Barrett et al., 2015) found four scales related to experiences of internal/external unity, noetic quality, sacredness; positive mood; transcendence of time/space; and ineffability.

Participants completed the MEQ at the end of each psilocybin session for a quantitative measure of the mysticomimetic experience, and scores from the first session were one of the factors taken into consideration when choosing the psilocybin dosage for each participant's second medication session. Participants who had MEQ subscale scores of < 0.6 (i.e., <60% of the maximum possible value on each of six subscales) for the first session, and whose therapists agreed that an increase would not be clinically contraindicated, were given the option to increase the dose for the second session. The determination that a higher psilocybin dose would be clinically contraindicated even though MEQ subscales scores were below the 0.6 mark would have been made on a case-by-case basis, and there were no such occurrences in the present study.

The primary measure of subjective psilocybin effects was the intensity subscale of the Hallucinogen Rating Scale (HRS; Strassman et al., 1994). The HRS was developed and validated in studies of DMT by Strassman et al. (1994), with further validation in users of ayahuasca (Riba et al., 2001), and has been used in studies of psilocybin in healthy volunteers (Griffiths et al., 2011). Mean and SD data for all subscales are presented in Bogenschutz et al. (2015). Here, we present the intensity subscale data for each participant.

The Altered States of Consciousness (ASC) rating scale was developed by Dittrich (1998) with further validation by Studerus et al. (2010), among others. While the original rating scale had five subscales, Studerus et al. (2010) performed an extensive validation study using the ASC to measure participant experiences of psilocybin, ketamine, MDMA and found 11 reliable subscales. The altered states of consciousness induced by psilocybin can be distinguished by the ASC, which shows good discriminant validity between states induced by psilocybin, MDMA, and ketamine (Studerus et al., 2010) and does not detect alcohol intoxication or the effects chlorpromazine as compared to placebo (Dittrich, 1998). Kraehenmann et al. (2017) found that one subscale, the blissful state scale, correlated with the presence of use of primary process language in descriptions of LSD sessions in healthy volunteers. Here, we present blissful state subscale data for each participant, as it may relate to the emergence of primary process thinking. See description of ego-dissolution coding below.

### Data analysis

Interview transcripts were analyzed using Atlas.ti qualitative data analysis software (Atlas.ti, 2004). Initial coding categories were established by known phenomena of interest such that coding was theory driven. This method is known as directed content analysis (Hsieh and Shannon, 2005). Coding categories included material related to alcohol/drinking, mysticomimetic experiences, ego-dissolution, interpersonal interactions, intrapersonal insights, motivation, commitment to change, music, stigma, the therapist/participant relationship, and difficult experiences during the psilocybin session. Categories were chosen because they reflected the phenomena of interest in the study (i.e., material related to drinking), reflected major areas of inquiry in the literature on change processes in psychedelic-assisted therapy (e.g., mysticomimetic experiences, ego-dissolution), or were noticed and suggested as potentially relevant by the co-authors either during the sessions themselves or after an initial reading of the transcripts. Transcripts were also categorized and compared depending on whether the transcript was from a first or second psilocybin session, and by whether or not the corresponding MEQ score met the 0.6 criteria described above.

The first author created coding categories and completed an initial coding of all transcripts, then presented a description of each of the categories and corresponding coded participant utterances to the co-authors for review. The third and fourth co-author, having conducted most and all of the debriefing sessions, respectively, and the second author, having coordinated the transcription process, were all familiar with the data set. All authors had access to the transcripts to verify and confirm the validity and completeness of coding. After reviewing the categories and coded material, the authors collaborated for discussion to ensure accuracy, completeness, and agreement. The first author reviewed the coding categories for keywords (words that frequently emerged in coded text for each code) and performed secondary searches of the data set using these keywords to identify passages that should potentially be included but were missed in the reading process, using judgment to determine final inclusion. After coding all transcripts, seven categories emerged as having substantial content for discussion. Here, we describe each category, including the theoretical background and our process for coding relevant participant utterances.

#### Mysticomimetic experiences

Previous research has demonstrated that, when administered in clinical research settings, psilocybin can precipitate mysticomimetic experiences that are personally meaningful (Griffiths et al., 2008). Strength of mysticomimetic experience has been correlated with positive outcomes in psychedelic research with healthy volunteers (Griffiths et al., 2006) and shown to mediate positive change in clinical populations (Garcia-Romeu et al., 2014; Griffiths et al., 2016; Ross et al., 2016), and a transformative spiritual experience has long been regarded as crucial to change by the traditional Alcoholics Anonymous philosophy (Forcehimes, 2004). With regard to the role of psilocybin-induced mysticomimetic experience in alcohol treatment, one possibility is that it results in increased motivation such that changes in drinking occur (Bogenschutz, 2013).

In order to code transcripts for each of the sub-domains of the MEQ, the items comprising each category in MacLean et al.'s (2012) factor analysis were reviewed and used to define coding categories for material related to each subscale. A thorough reading of the three transcripts from sessions in which the participant met the >0.6 MEQ score cutoff on all subscales was then performed, with the researcher coding participant utterances that articulated each category. The category of ineffability is somewhat ironic in a study of people's words; actually, people often say they cannot describe mystical experiences but go on to provide detailed verbal accounts (Yaden et al., 2016). In the present study, we coded participant descriptions of the sense that the experience was too difficult to describe as examples of ineffability.

#### Ego-dissolution

Falkenstrom (2003) wrote that what is meant by the self in psychoanalytic thought is distinct but not incompatible with the self as understood in Buddhist psychology. In psychoanalytic thought, the ego is the part of the personality that holds one together, balancing instinctual drives and moral rules, and a strong, healthy ego results in psychological health (Falkenstrom, 2003). From the Buddhist perspective, mental suffering results from the erroneous belief in ego or self as an enduring, unchanging entity with inherent existence (Van Gordon et al., 2017). Generally, when psychedelic researchers speak of ego-dissolution, they are referring to a loosening of the latter concept, which results in new insights and diminished psychological suffering. Hence, experiences of ego-dissolution, which could be considered pathological from the Freudian perspective, might be healing from perspective of Buddhist psychology. This concept is also discussed as the experience of *not-self* or *emptiness* in Buddhist psychology (Van Gordon et al., 2017) and may be described as a *loss of self-boundaries* or *altered sense of self* in the fields of psychology and psychiatry.

Global connectivity between brain structures and functional networks is increased by LSD, and the strength of these alterations are positively correlated with the sense of ego-dissolution; a sense of the self as not separate from others, or even as a distinct individual bound by time and space (Tagliazucchi et al., 2016). Psilocybin has specifically been found to decrease metabolic activity in the default mode network (DMN; Carhart-Harris et al., 2012), a network of neural structures related to self-referential thinking, autobiographical memory, and planning (Buckner et al., 2008), which may further contribute to the experience of ego-dissolution. More recently, Lebedev et al. (2015) found that psilocybin induced the perception of ego-dissolution, or loss of a sense of “I,” by decreasing functional connectivity in medial temporal lobe and high-level cortical regions, decreasing integration of the salience network, and decreasing communication between hemispheres. In order to code transcripts for descriptions of ego-dissolution we created a coding category with the same title and selected examples of participants mentioning loss or change in their sense of self, identity, or “I.”

Primary process and secondary process thinking are thought to reflect Freudian theory of the pleasure and reality principles, the “Id” and “Ego” respectively, with primary process thinking becoming more pronounced in altered states of consciousness with corresponding reduction of activity in the DMN, a secondary process function (Carhart-Harris et al., 2014; Kraehenmann et al., 2017). Recently, a guided mental imagery task used to enhance recall of LSD and placebo sessions in a structured way and produced measurable differences in primary process thinking (Kraehenmann et al., 2017). Furthermore, primary process thinking reflects emotional processes which are a key factor in the subjective experience of psilocybin-assisted therapy (i.e., Belser et al., 2017), perhaps due to reduced amygdala reactivity and increased positive mood in healthy volunteers (Kraehenmann et al., 2015). Kraehenmann et al. (2017) found that the blissful state subscale of the ASC rating scale correlated with the use of primary process language in descriptions of LSD sessions in healthy volunteers. Here, we include blissful state subscale data for consideration alongside statements that reflect experiences of ego-dissolution and the emergence of primary process thinking.

#### Relationship to alcohol

This category, originally titled *insights about drinking*, was populated with content about drinking gathered by auto-coding interviews for the words *drink, drinking*, and *alcohol*. Additional passages that referred to drinking and alcohol but did not use these words were added as identified. As expected given that the study protocol calls for the therapists to discuss the relationship of the psilocybin experience to drinking during debriefing, all transcripts contained some mention of this topic. Upon review of coded content it was noted that participants consistently described a change in their relationship to alcohol, prompting a modification in the category title to more accurately characterize this theme.

#### Motivation for change

Motivation for change has been clearly delineated as a factor correlating with success in changing addictive behaviors, and psilocybin may enhance motivation for change in alcohol use through its subjective experience or via a more basic biological mechanism. For instance, psilocybin's anti-addictive properties (Ross, 2012) may result in improved self-efficacy relative to abstinence. In the present study, one of the therapists conducted MET, which incorporates the principles of motivational interviewing, to increase the participant's internal motivation to make positive, healthy behavior changes. Change talk—specific sentences and phrases in which people express confidence in their ability to change (self-efficacy) or discuss reasons or desire to change—can be identified in debriefing sessions. In order to code material for this category, we created a coding category for *confidence, motivation, & resolve* and selected participant speech indicative of motivation for change such as expressions of readiness for change, willingness to change, and the importance of change.

#### Commitment to change

Self-efficacy, desire, and reasons for change are important precursors to behavioral modification, but are distinct from instances of strong commitment to make a change. In order to analyze transcripts for examples of commitment language, we coded the use of key commitment phrases provided in the MET manual. We found some overlap with expressions of confidence, reasons, and desire described above; however, commitment language is distinguished because it is a direct mention of a plan or intention to behave differently.

#### Dysphoric experiences and their resolution

Recent survey research has shown that dysphoric experiences while using psilocybin in non-clinical settings are often associated with lasting increases in wellbeing (Carbonaro et al., 2016). All study participants were prepared for the possibility of dysphoric experiences during their preparatory sessions, and given specific guidance and tools for managing them. For instance, participants are taught to use breath awareness to stay centered, and guidance to approach dysphoric experiences with curiosity and openness instead of resisting these occurrences. Additionally, antihypertensive, anxiolytic, and antipsychotic medications were on hand for emergency treatment. These were not needed at any point in the study; however, participants could be reassured by their availability. To code for dysphoric experiences, a coding category was created for material expressing fear, anxiety, or other distressing emotional or physical experiences during the psilocybin session.

#### Stigma

Stigma—an association of shame, disgrace, or social disapproval—is a compound issue for psychedelics as potential treatments of addictive disorders. First, stigma is a major barrier to engagement in any addiction treatment, including conventional methods (Corrigan et al., 2006; Luoma et al., 2007). This stigmatization of addiction even extends to providers of alcohol and drug treatment services (Eaton et al., 2015). Second, psychedelics are stigmatized medicines by nature of their contentious history and current legal status. Psilocybin-assisted treatment for alcohol dependence is therefore a stigmatized treatment for a stigmatized disorder. Understanding how patients experience both of these stigmas will aid in minimizing their roles as hindrances to the future availability and use of psychedelic-assisted treatment. Mentions of stigma, either experienced or anticipated, were coded by reading all transcripts for relevant material such as reporting worry about telling others, avoiding telling others, or anticipating negative reactions from others.

## Results

Debriefing session recordings totaled 899 min and included two sessions from seven participants and one session from each of another three participants. The fourth author conducted all sessions, 12 with the third author as co-therapist, and five with a physician co-investigator as co-therapist. Table 1 presents participant demographics with psilocybin doses and change in % heavy drinking days, HRS intensity scale, blissful state scale data for each participant. As previously reported, intensity scale mean was 2.43 (*SD* 1.03) in 1st sessions with dose of 0.3 mg/kg (*N* = 10), and 2.0 (*SD* 1.14) in 2nd sessions with dose of 0.4 mg/kg (*N* = 6). Only one of the seven participants, Adam, did not increase the dose to 0.4 mg/kg for the second session. Adam's intensity score of 3.5 for the first session was >1 *SD* above the mean, and he was the only participant to score >0.6 on all of the subscales of the MEQ for his first session, therefore an increased dose was not warranted.

**Table 1:** Participant demographics, intensity, and blissful state scores.

**Name**	**Age**	**1st Session dose^¤^**	**Intensity**	**Blissful state**	**2nd Session dose^¤^**	**Intensity**	**Blissful state**	**Reduction in heavy drinking days^§^**
Jason	42	0.3	3.00	27.67	–	–	–	**100%**
Adam	47	0.3^u^	3.50	52.67	0.3^u^	3.75	16.33	**100%**
Ashley	41	0.3	2.25	78	–	–	–	9.21%
Charles	30	0.3	2.25	13.67	0.4	2.50	4	19.43%
Zach	25	0.3	2.75	44	0.4	1.75	14.33	72.26%
Keith	40	0.3	1.75	12.33	0.4	1.25	3.67	42.84%
Lisa	56	0.3	2.25	18	–	–	–	X
Heather	28	0.3	0.00	3.33	0.4	0.25	3.17	**100%**
David	53	0.3	3.00	18	0.4	3.25	81.33	13.67%
Michelle	39	0.3	3.50	36.67	0.4^u^	3.00	92	**100%**

¤ *Dosages reported are mg of pure psilocybin per kilo of the participant's weight. Actual dosages were compounded prior to the medication session based on current weight*.

*–Participant did not complete a second psilocybin session*.

u *Indicates scores >0.6 on all MEQ subscales as rated after each session. If this criterion was met during the first session then psilocybin dosage was not increased for the second session*.

§ *Relative proportion of the number of heavy drinking days reported during weeks 9–12 of the study to the number of heavy drinking days at baseline. For instance, if a participant reported 30 drinking days at baseline and 6 during weeks 9–12, they have a reported a reduction of 80%. For full analysis of this and other outcomes see Bogenschutz et al. (2015)*.

*1st session with dose of 0.3 mg/kg (N = 10) HRS Intensity score Mean 2.43 (SD 1.03) Blissful State Mean 30.4 (SD 22.7)*.

*2nd Session with dose of 0.4 mg/kg (N = 6, underlined scores) HRS Intensity score mean 2.0 (SD 1.14) Blissful State Mean 33 (SD 41.8)*.

*X—No data available*.

### Mysticomimetic experiences

MEQ subscale and total scores for each participant/session are reported in Table 2. The following participant expressions, drawn from the three sessions in which participants scored >0.6 on all subscales of the MEQ, illustrate the phenomenon of internal/external unity, noetic quality, sacredness; positive mood; transcendence of time/space; and ineffability.

**Table 2:** MEQ scores.

	**1st Session**							**2nd Session**						
	**Unity**			**Transcendence**	**Positive mood**	**Ineffability**	MEQ total^*^	**Unity**			**Transcendence**	**Positive mood**	**Ineffability**	MEQ total^*^
	**Unity**	**Sacred**	**Noetic**					**Unity**	**Sacred**	**Noetic**				
Jason	0.80	0.71	0.65	0.40	0.40	0.52	0.54	–	–	–	–	–	–	–
Adam	0.73	0.74	0.75	0.74	0.74	0.72	0.77	0.83	0.74	0.90	0.95	0.66	1.00	0.84
Ashley	0.40	0.43	0.80	0.57	0.57	0.64	0.52	–	–	–	–	–	–	–
Charles	0.33	0.31	0.40	0.54	0.54	0.60	0.42	0.10	0.17	0.10	0.50	0.26	0.28	0.25
Zach	0.27	0.49	0.40	0.60	0.60	0.52	0.44	0.13	0.23	0.15	0.18	0.40	0.32	0.24
Keith	0.27	0.20	0.25	0.17	0.17	0.32	0.24	0.20	0.14	0.35	0.13	0.20	0.28	0.20
Lisa	0.77	0.57	0.75	0.49	0.49	0.68	0.69^*^	–	–	–	–	–	–	–
Heather	0.00	0.00	0.00	0.03	0.03	0.00	0.02	0.00	0.00	0.00	0.00	0.06	0.00	0.01
David	0.37	0.43	0.75	0.57	0.57	0.52	0.50	0.70	0.71	0.80	0.48	0.86	0.72	0.70^*^
Michelle	0.67	0.66	0.90	0.49	0.49	0.76	0.61^*^	0.90	1.00	1.00	0.80	0.97	0.92	0.92

* *Indicates total score over 0.6 with at least one subscale that did not meet the 0.6 criterion*.

*–Indicates participant did not complete the second psilocybin session*.

*2nd Session with dose of 0.4 mg/kg (N = 6, underlined scores)*.

#### Internal/external unity, noetic quality, sacredness

It was just this feeling of oneness. (Adam, Session 2)

And, it really was this freedom of knowing … that bigger ultimate place to be…I got that in a way that I had never understood it before. Like, it was just—I could feel it, you know? I could see it, I could sense it, I could hold it…And it just became a whole new, you know, realm and understanding, and I thought—“and I want to know that more. I want that every day, and I want it to be a part of my life every day.” You know? It was just such a peaceful sense of, “It doesn't even matter what else happens.” (Michelle, Session 2)

#### Positive mood

I felt peaceful. I feel more invigorated. And I feel like there's something else in life. (Adam, Session 2)

#### Transcendence of time/space

Changes in perception of time and space, phenomena that seem stable and predictable in ordinary states of consciousness, may be awe inspiring when experienced during a psilocybin session. Adam reported shifting spatial perspectives, which, while not necessarily indicative of transcendence of space, do indicate change in spatial perception.

It was like infinity almost. Everything was kind of a sense of infinity.…You know it was like it was open. And I was going in and out of it. And I was in the middle of it. You know, all this space. It was kind of like looking up and a dome was over me. Or sometimes it would be below me. (Adam, Session 2)

#### Ineffability

Adam, who most directly expressed that his experience was ineffable, discussed how he felt he could express his experience better through visual art than words.

A painting's going to explain it better than words can. Just my personality, I just have a hard time putting spiritual… I mean it was spiritual for sure.

Finally, Michelle touched on several aspects of a mysticomimetic experience in discussing the possibility of having experienced a direct connection with God. The following exchange between her and her therapists illustrates how this came to light in the debriefing session.

Therapist 1: One of the things you said yesterday about the more spiritual aspect of the experience was that you felt this really direct connection with God. Presence of God. In a more personal, immediate way, than…

Michelle: Yeah, in that way, it was like, to me, it was like, “Start here. Start right here,” you know, “Take this gift of love, salvation, connection, and everything else will come.” You know? “Make this first,” you know, “Make this the prevalent thing in your life, and everything else will come.” That's what I felt in all aspects of my life, you know? And I believe that.

Therapist 1: That that's really the central, core thing…

Michelle: Mmhmm. That is the center. And I know that, I've known that, but I know it in a different way, you know?

Therapist 2: You really feel like you got that message…

Michelle: Yeah, it's…I enjoyed it. I enjoy that, you know? It makes me feel good. It makes me feel connected again. And not, flailing out there somewhere. Yeah. It's a purpose, you know? It's a purpose. And that's the whole thing is, I've been living life really without my own personal purpose, which should be my relationship with God, you know? I've– it's just been an unpurposeful life. Regardless of, you know, “everyone looks fine,” and “everyone's do–,” but it's been without a true purpose. And I don't know if the drinking allowed me to not pay attention to that, you know? I didn't have to think about it or how I was gonna work my way out of whatever it was in my own head, or…But that's all I can imagine. Because I didn't get all lost in what happened, you know? [laughs] I mean, you feel good for the first 5 min, then it's like, “same thing.”

### Ego-dissolution

Table 1 presents the blissful state subscale of the ASC (Studerus et al., 2010) which has been found to correlate to LSD induced increases in primary process thinking—and corresponding reduction in secondary process thinking, of which ego is a function (Kraehenmann et al., 2017). Although all transcripts were inspected for mentions of ego-dissolution, 10 of the 13 observed instances were found in transcripts from sessions correspond to high MEQ scores, suggesting there is also a relationship between scores on the MEQ and the experience of ego-dissolution. Adam, the only participant who reported high scores across all four factors of the MEQ in both psilocybin sessions, described an experience of ego-dissolution during his first session that included reference to a fear of completely losing his sense of self, and taking comfort in retaining that identity.

I guess you know it was a good feeling when I, you know I'd say “okay I'm here for a reason.” I'm still Adam. You know, it's funny because my friend who's a psychologist … He sent me an email, he said “let go, bon voyage,” was his email and I read it afterwards, but I do have a problem letting go. I try to tell myself over and over but then on the other hand, I didn't I guess I wanted to still remember who I was… I didn't want to “lose it” … I guess because I still have fear, I mean I do have a lot of fear…it still felt good that I knew who I was. Because I think if I would've, I shoulda let go more…I did let go and enjoy it, and I did that in parts, but I don't know how much I let go because I still enjoyed the part knowing it helped me to know that I was still Adam. And I was here… I think I maybe would've had a bad or worse experience if I didn't remember who I was.

During the discussion of the experience with Therapist 1 he was again reminded of the need to balance an intention to allow ego-dissolution to occur, with not forcing it to happen.

Therapist 1: The standard advice is to really let go and surrender to the experience as much as possible, but you can't force that, and so you don't want somebody else pushing you off the cliff, and you don't want to push yourself off the cliff that you don't feel ready to jump off.

In his second session Adam reported the following.

There was a part there where I felt gone. … it wasn't like last time where I always remembered I was Adam. There was a part there where I was gone. … I would come back and you know, I remember telling myself you took this to try and help. And it is helping. … There's the old Adam, then the new Adam without alcohol.

Michelle, who had high MEQ ratings in her second psilocybin session, stated the following in relation to her experience of herself during the session.

I was breaking up into little droplets of water, and then coming whole again, and…And it was really pretty. It was, like, on a wave … It felt like being a baby, you know? Just rocking. And I'm thinking, “This is nice, this–there's just, sort of, weightless.”

### Relationship to alcohol

Therapists asked participants about how their psilocybin experience related to their drinking. Here, several describe changes in their personal relationship to alcohol.

I just thought yesterday was really good for identity, and it kind of secured how I've been feeling for some years, and, it made it more stable. I'm still gonna have a lot of challenges; I don't think so much of drinking as much, because I know I have to be clear minded a lot more. And, uh, those are things that just sidetrack me. So, it, believe it or not, you know, it benefited me. It was medicinal. It wasn't something recreational where I just stayed there laughing. So, you know, it helped. It did help. It's kinda…It's gonna help me, just, go forward and remember, you know…the insight that I got was–it was worth it. You know? It was really deep. (Jason, Session 1)

I had the series of, “you did this, you did this, look at this, this, this and this with the liquor,” then I saw this long tunnel and I was expecting to see a light but it didn't quite happen. I didn't see the light at the end of the tunnel, but I did see like chains; and the significance to me was that I was in bondage by the alcohol. (Lisa, Session 1)

Adam, an artist, discussed a change in the relationship between carving sculptures and drinking.

Like say it was October or September 14', I'm just throwing out a date, after I had been back drinking a week, it was like I had been drinking for a year again. Okay, I'm going to carve again I'm going to drink, they go hand in hand. Or whatever I'm going to do my art I'm going to drink, just a matter of maybe holding out until noon, or trying to hold off until noon and being happy to make it until 10. But right now it's just a different mentality. So when I am [drinking] art and alcohol go together. But today it's not like that. (Adam, Session 1)

### Motivation for change

These expressions can be understood as examples of change talk and related to the motivational enhancement aspect of the META treatment.

I feel pretty confident… I haven't wanted [alcohol], I guess I kind of feel reinvigorated since before the session. (Adam, Session 2)

I feel more confident in saying, “You don't have to pass [me drinks], guys,” and I don't feel guilty about that where I typically would. (Charles, Session 1)

I can change this. Right now. Today, in the moment, right then and there. (Michelle, Session 1)

I feel uh you know, the only reasons I want to quit … my art sometimes suffers from drinking and, so I feel inspired to do my art and, you know, work maybe until tonight, you know, and just work on my art and not have to you know, drink. (Adam, Session 1)

I feel more positive about being able not to drink. I know there will be those times there's a craving and it's there and the opportunity to do it but then maybe having a chance to tell myself … bring out the tools from the session that I felt and see if they work, try to use those. (Zach, Session 1)

### Commitment to change

The following instances demonstrate commitment to change, consistent commitment sought in the MET therapy.

I'm going to do something different, you know? I'm going to do it different. (Michelle, Session 2)

If those urges come up I plan on fighting them (Adam, Session 1)

I want to serve my purpose. I want to find my mission. I want to do it right, and I'll do it the best I can, you know. (Jason, Session 1)

If I really felt like if I really wanted to drink I would… reach out to some people. I know that much, you know. (Adam, Session 2)

I will do my best not to fan the fires. (Charles, Session 1)

### Dysphoric experiences

The following statements illustrate how participants described dysphoric states during the psilocybin session, and often balanced their descriptions with comments about the meaningfulness of their struggles.

There was some darkness involved in some of it, but I could understand through the darkness that I wasn't alone (Jason, Session 1)

I was having a hard time, and I was crying. That's when my grandma was calling me, and rocking me, and singing to me. So that brought up a lot of emotions. (Ashley, Session 1)

That whole experience just felt for me like, a fever nightmare. (Charles, Session 2)

It was actually the music and I guess the effect of the drug that was bringing out the, the negative… and I was kind of wondering, “hmm, I guess that's the intent, maybe,” in the bizarre music… (Lisa, Session 1)

Yesterday was devastating, but it was the kind of devastating that I could wake up from knowing and feeling everything as though it had happened, but I still have everything. It happened like it was supposed to happen. … I was super hot, I was rolling into the crack of that couch. I could have used a fan, but all that torture I felt was just part of the … If I was uncomfortable, I deserved it. That's how I looked at it. … I was always aware of what was going on, and I knew that this was completely not what I had planned or expected but it was happening, and I needed to take full advantage of it as gut-wrenching as it was. (Michelle, Session 1)

### Stigma

Here, three participant quotes illustrate the difficulty participants had in sharing their experiences with those who are close to them. The first illustrates a case in which Adam perceived that a family friend would dismiss psychedelic-assisted treatment as just another form of addictive behavior.

I don't think he'd be too keen on the study, because I know how he is about, he calls it the marijuana maintenance program, a lot of people quit alcohol and go to pot, and …my wife actually just wishes he would smoke pot. But my sponsor he doesn't really believe that, he seen them creep back to it with the [marijuana] maintenance program, so he's not real big on that. (Adam, Session 1)

Zach discussed how the unique character of psychedelic-assisted therapy, combined with his difficulty in describing the psychedelic experience, made it hard to convey to a loved one how it could be helpful.

I talked to her and let her know what I was thinking and she kind of related, but she of course thought it must have felt crazy, sitting in a room tripping out and losing your sense of; your senses. Altogether, it's hard to just say it in words anyways. (Zach, Session 1)

Michelle thought she might just keep the experience to herself, as she was unsure that others could comprehend it.

My sister's the only one that knows that I've kind of gone through this. And I've told one friend a little bit, and, um…You know, I don't know that I'm actually going to openly express this. I don't know if I'm ready to, you know what I mean? … I don't know that anyone could understand it. … I'm not ready to really talk a whole lot about it to others. (Michelle, Session 2)

## Discussion

The present analysis illustrates a variety of concepts in psilocybin-assisted treatment of alcohol dependence and demonstrates that there may be several change-related phenomena of interest, as opposed to one key factor prompting change. Some of these phenemona have been previously explored at length in the literature and others are fairly novel topics in the realm of clinical psychedelic research. Mysticomimetic experience, for instance, was one of the first concepts to be explored and described in clinical research on psychedelics and remains a topic of enduring interest (i.e., Pahnke, 1969; Doblin, 1991; Griffiths et al., 2008; MacLean et al., 2011). We expect continued focus on this topic as it has been shown to account for some, but undoubtedly not all, of the positive mental health outcomes of psychedelic therapy. The excerpts presented here primarily come from sessions with high MEQ scores, yet there were many debriefing sessions in which participants did not clearly articulate the component phenomena of mysticomimetic experiences. This may be for a variety of reasons including that they may have already discussed the relevant material at end of their psilocybin session, memory of the experience may have shifted since completing the MEQ the day before, or these phenomena may not have been the most prominent or interesting to the participants themselves. Additionally, doses may have been below the level needed to induce such experiences, as indeed they were detected in only 3 of the 17 sessions by the MEQ. Finally, we note that the effects of psilocybin are measurably distinct and unique to classic hallucinogens, as demonstrated by prior research using the ASC and MEQ scales.

Stigma, on the other hand, is a relatively under-explored topic in psychedelic clinical research, although it has been addressed in the context of traditional use of psychedelics and with regard to general stigma faced by those who have or treat addictive disorders. Likewise, there is extensive literature on MI and the type of changes it produces in addictive behaviors, however it had not previously been tested in the context of psychedelic therapy. The present treatment model is specifically designed so that clinicians who are already trained in the empirically supported MI approach can collaborate with a therapist who has the training to provide the psychedelic preparation and integration sessions to form a co-therapist dyad. The samples of change and commitment talk illustrated here demonstrate that they do occur in the present treatment model when therapists are trained to elicit and respond to it.

Other qualitative studies of participants in psychedelic-assisted treatment trials for smoking cessation (Noorani, 2017), cancer anxiety (Belser et al., 2017), depression (Watts et al., 2017), and psychostimulant abuse (Schenberg et al., 2017) have involved interviewing participants about their experiences several weeks to months afterward. In contrast, this analysis used actual clinical sessions as the data set. Our approach avoided the creation of a separate situation and the introduction of interviewer dynamics, but limited our ability to ask participants about specific constructs of interest, such as emotional content, that might not have been mentioned in the debriefing session. Furthermore, participants may disclose different things to their therapists than they would to an interviewer. While analysis of clinical sessions may give a clearer picture of what actually occurs in treatment, and an interview may give a clearer picture of how the participants describe and think about what occurred in treatment. We believe both approaches can complement each other in advancing our understanding of the change process in psychedelic-assisted therapy. Authors EN and MB are initiating a qualitative interview study of participants in the ongoing RCT of psilocybin-assisted treatment of alcohol dependence.

Another potential confound in the analysis of debriefing sessions completed the day after a psilocybin session as in this study, or qualitative interviews conducted several months later as in several of the aforementioned studies, is alteration in recollection of the experience due to delayed recall. Linton et al. (1964) investigated this phenomenon by asking subjects about the presence or absence of the same set of 74 possible qualities of their experience both during and the day after an LSD or placebo session. They determined that participants described their experiences differently the day after, tending to endorse fewer overall items by adding fewer items than they dropped. The most stable items, those consistently reported both during and after the session, tended to relate to the most profound effects of LSD. With regard to endorsing new items the following day, the authors suggest that subjects may have endorsed items afterward that they were in denial of or afraid to acknowledge during the LSD experience. In the present study, we note that the therapists may have assisted with recall by reminding the participant and asking about statements or observations made during the session, but did not employ any formal recall-enhancing techniques. In the therapeutic task, accuracy of recall is, in and of itself, less important than how the patient remembers, understands, and integrates the experience.

Finally, in reviewing the HRS intensity, ASC blissful state, and MEQ scores we note that Heather had scores of 0 or very low on most scales for both sessions. These scores reflect an extremely mild subjective experience, and indeed Heather indicated in her debriefing sessions. Her 100% reduction in heavy drinking days, however, indicates she did change her drinking during the study. This may be due to the MET treatment working alone, anti-addictive neurobiological effects of psilocybin, or be a coincidence, anomaly, or measurement error. Future and larger studies may clarify how and why certain individuals such as Heather achieve changes in drinking without experiencing substantial subjective effects of psilocybin.

## Limitations

The findings presented herein represent an analysis of 17 debriefing sessions transcribed from recordings made during the pilot-feasibility study of psilocybin-assisted treatment of alcohol dependence at University of New Mexico between 2012 and 2013. Because these documents reflect actual clinical sessions there is considerable variation in their length, the content and topics, and even the format—with several having been conducted over the phone and several with only one therapist present for all or part of the session as opposed to the usual two. Our analysis did not differentiate or compare sessions based on these factors. Furthermore, the transcriptions were completed by four graduate assistants (none of whom were study therapists) and likely varied in quality and accuracy.

Codes were selected and defined based on consensus between authors as opposed to predefined, standard criteria, and should be replicated in a future study. Also, because the study did not rely on a semi-structured interview, use of any formal recall task, such as guided imagery, was not included. Future studies in which the aim is to obtain uniform data for content analysis could include a formal recall task in the debriefing session or interview to elicit a more consistent data set.

## Author contributions

EN proposed the structure, rationale, and research methods for the present qualitative analysis, coded, and analyzed the data, wrote the first draft of the manuscript, and coordinated revision efforts between all authors. DM coordinated data collection and transcription and collaborated the design of the present analysis and manuscript writing. AF was a therapist on the clinical trial from which the data were collected and provided input in the data analysis and manuscript revision. MB was the PI and a therapist on the clinical trial from which the data were collected and provided input in the data analysis and manuscript revision.

### Conflict of interest statement

The authors declare that the research was conducted in the absence of any commercial or financial relationships that could be construed as a potential conflict of interest. The handling Editor declares a past co authorship with authors MB and AF.
